# Response of blood glucose and GLP-1 to different food temperature in normal subject and patients with type 2 diabetes

**DOI:** 10.1038/s41387-022-00208-0

**Published:** 2022-05-27

**Authors:** Yun Hu, Peng Zhang, Bo Ding, Xin Cao, Yi Zhong, Kok-Onn Lee, Jian-Hua Ma

**Affiliations:** 1 Department of Endocrinology, Wuxi People’s Hospital Affiliated to Nanjing Medical University, Nanjing, China; 2 Department of Endocrinology, Nanjing First Hospital, Nanjing Medical University, Nanjing, China; 3 Department of Endocrinology, National university hospital of Singapore, Singapore, Singapore

**Keywords:** Type 2 diabetes, Nutrition

## Abstract

**Background:**

Eating behavior is a major factor in type 2 diabetes. We investigated the different responses of glucose-regulating hormones to cold and hot glucose solutions in normal subjects and patients with type 2 diabetes.

**Methods:**

In this crossover, self-controlled study, normal subjects (*N* = 19) and patients with type 2 diabetes (*N* = 22) were recruited and randomly assigned to a hot (50 °C) or a cold (8 °C) oral glucose-tolerance test (OGTT). The subsequent day, they were switched to the OGTT at the other temperature. Blood glucose, insulin, GIP, glucagon-like peptide-1 (GLP-1), and cortisol were measured at 0, 5, 10, 30, 60, and 120 min during each OGTT. After the hot OGTT, all subjects ingested hot (>42 °C) food and water for that day, and ingested food and water at room temperature (≤24 °C) for the day after cold OGTT. All participants had continuous glucose monitoring (CGM) throughout the study.

**Results:**

Compared to cold OGTT, blood glucose was significantly higher with hot OGTT in both groups (both *P* < 0.05). However, insulin and GLP-1 levels were significantly higher in hot OGTT in normal subjects only (both *P* < 0.05). The GIP and cortisol responses did not differ with temperature in both groups. CGM showed that normal subjects had significantly higher 24-h mean glucose (MBG) (6.11 ± 0.13 vs. 5.84 ± 0.11 mmol/L, *P* = 0.021), and standard deviation of MBG with hot meals (0.59 ± 0.06 vs. 0.48 ± 0.05 mmol/L, *P* = 0.043), T2DM patients had higher MBG only (8.46 ± 0.38 vs. 8.88 ± 0.39 mmol/L, *P* = 0.022).

**Conclusions:**

Food temperature is an important factor in glucose absorption and GLP-1 response. These food temperatures elicited differences are lost in type 2 diabetes.

## Introduction

While the timing [1] and frequency of meals [2, 3] have been noted as important factors for the prevalence of diabetes and blood glucose control, food temperature has not been seen generally as important in type 2 diabetes mellitus (T2DM). Recently, ambient food temperature has attracted some attention in the understanding of diabetes in Asian populations—where food temperature had some effects on the glycemic response to rice [4] and potatoes [5]. Early phase insulin release in animal studies (anesthetized rats) [6] was also reported to be affected by food temperature. Increased food temperature may activate the nerve system via several heat-activated ion channels, such as TRPV1 [7]. However, as far as we know, there have not been comparisons of the endocrine effects of ambient glucose or food temperature on the response to OGTT, or to normal daily meals.

Previous studies showed that gastric emptying was positively correlated with blood glucose in the first 30 min after glucose ingestion, and was inversely related to blood glucose at 120 min [8]. Gastric emptying may also influence GLP-1 release [9]. The cold and hot ambient temperature of ingested food has been shown to slow gastric emptying via affecting antropyloroduodenal motility and gastric electrical activity [10, 11], but as far as we know, the subsequent effects on blood glucose, and the responses in insulin and other gut-related hormones, have not been studied. There are large differences in ambient food and drink temperatures between different cuisines in most countries. Cold drinks are common in fast-food restaurants, but hot soups are the norm in many meals in much of East Asia. In recent years, there has been a large increase in many parts of East Asia, especially in China, of “hot pot” food, served often at temperatures above 50 °C.

In this study, we investigated the blood glucose, and glucose-responsive hormones to a standard oral glucose-tolerance test (OGTT) with hot or cold temperature glucose in normal subjects, and in newly diagnosed untreated patients with T2DM. We further performed continuous glucose monitoring (CGM) in these two groups on the two different days, to compare the glucose profiles of hot versus cold food and water throughout the day.

## Materials and methods

### Study population

We enrolled 19 normal subjects and 22 newly diagnosed patients with T2DM at the Nanjing First Hospital between June 2017 and October 2019. The normal subjects were healthy and had no known illness. The patients with T2DM were all newly diagnosed through health screening and had mild diabetes that did not require urgent treatment. The inclusion criteria for the two groups were as follows: (1) normal participants: no previous history of diabetes or any other illness; fasting plasma glucose <6.1 mmol/L and 2-h plasma glucose <7.8 mmol/L after 75-g OGTTs (the diagnostic criteria of World Health Organization 1999); (2) newly diagnosed patients with T2DM (diagnostic criteria of World Health Organization); HbA_1c_ <86 mmol/mol (10.0%). Both groups had BMI of 18.0–28.0 kg/m^2^ and were aged 18–60 years. Participants with the following were also excluded: (1) any history of hypoglycemic agent intake or were presently taking hypoglycemic agents; (2) any abnormality in liver or kidney function on blood and urine investigations; (3) any history of systemic corticosteroid use in 3 months; (4) any recent infections or acute medical events; (5) pregnancy.

The study was reviewed and approved by the Institutional Ethics Committee of Nanjing First Hospital, Nanjing Medical University, and signed informed consent was obtained from all study participants. This trial was registered in ChiCTR (ChiCTR-OOC-17011643, registered June 12, 2017, http://www.chictr.org.cn/showproj.aspx?proj=19708). This study was carried out in accordance with the Helsinki Declaration, and all methods were carried out in accordance with relevant guidelines and regulations.

### OGTT with different temperature

All experiments were conducted at a dedicated facility at the hospital with an ambient temperature of 22 °C. After an overnight fast (>10 h), half the participants were assigned randomly to either a hot or cold OGTT at 8:00 am, and instructed to take the corresponding hot or cold food throughout the day. The participants then all took the alternative temperature OGTT and food the next day.

Hot and cold glucose solutions were prepared as follows: 75 g anhydrous glucose was dissolved in 300 ml of water. The cold glucose solution was cooled in a refrigerator (4 °C), while the hot glucose solution was heated in a water bath (55 °C). The final temperature of the glucose solution was measured just before oral administration to ensure the temperature of 6–8 °C for the cold OGTT and about 50 °C for the hot OGTT. Venous blood was sampled at 0, 5, 10, 30, 60, and 120 min during OGTT. The OGTT was repeated at the opposite temperature the next morning. After the hot OGTT, participants were given hot food for lunch and dinner for the rest of the day and instructed to avoid cool food or water. Similarly, after the cold OGTT, the participants took cold food and water. The food on both days was identical in composition and total calorie content to avoid differences in “chili” (capsaicin) related and other gustatory differences. The “hot” foods included hot soup, noodles, rice, vegetables, and meat at temperatures of 45–55 °C. Cold food was at room temperature of 20–24 °C. None of the subjects reported any difficulty with these food temperatures. The lunch time was from 11:00 to 12:00, and supper time was from 17:00 to 18:00. All meals were consumed within 30 min.

### Clinical and laboratory assessments

Height, weight, age, and medical history were collected at the first visit. Body mass index (BMI) was calculated as weight divided by the square of height (kg/m^2^). Plasma blood glucose levels were measured using the glucose oxidase method with an auto-analyzer (Modular E170, Roche, Mannheim, Germany). HbA_1c_ was measured by high-performance liquid chromatography assay (Bio-Rad Laboratories, Inc., CA, USA). Insulin levels were measured by chemiluminescent immunometric assay using the Modular Analytics E170 (Roche^®^ Diagnostics GmbH, Mannheim, Germany, reference range: 2.3–11.6 mU/L). Plasma cortisol was determined with quantitative radioimmunoassay (Beijing North Institute of Biological Technology, CN). Measurements of glucagon-like peptide-1 (GLP-1, 7–36 and 7–37—amide) and glucose-dependent insulinotropic peptide (GIP) were made in blood collected in precooled specimen tubes with added protease and DPP-IV inhibitors, and by ELISA assays (USCN LIFE, CN; intra-assay precision: CV < 10%; interassay precision: CV < 12%).

### Continuous glucose monitoring

All participants had CGM for 3 consecutive days during this study. A CGM (Medtronic Incorporated, Northridge, CA, USA) sensor was implanted in the anterior abdomen one day before the first OGTT. Participants were instructed in the use of the device and were asked to measure capillary blood glucose four times daily for calibration. Interstitial glucose was continuously measured every 5 min. The CGM sensor was removed 24 h after the second OGTT. The participants were asked to maintain their activities without strenuous exercise during CGM for the three consecutive days. Patients with capillary blood glucose ≥20.0 mmol/L at any time point were removed from the study. The 24‐h mean glucose (MBG), standard deviation of MBG (SDBG), coefficient of variation (CV), 24‐h largest amplitude of glycemic excursion (LAGE), and time in range (TIR) were recorded and compared between the two temperatures in the two groups [12, 13].

### Statistical analysis

All statistical analyses were performed using the SPSS 22.0 software (SPSS Science, Chicago, USA). All variables were tested for normal distribution of the data and were expressed as mean ± SEM, or as median (IQR). Each of the variables: glucose, insulin, GLP-1, GIP, and cortisol were analyzed by ANOVA for repeated measurements to test for statistical significance of the differences between hot and cold OGTT. If there was statistical significance between the two temperatures (*P* < 0.05), this was followed with a Bonferroni test for specific time points. The paired *t*-test was used in the comparison of CGM data between cool and hot meals in the same subject. Differences between the groups were examined using the Student’s unpaired t-test or Mann–Whitney *U* test. All comparisons were two-sided at a 5% significance level. A *P* value < 0.05 was considered statistically significant.

We tested the cold and hot OGTT in six normal subjects in a pre-study. The area under the curve (AUC) of blood glucose after glucose administration was 241.67 ± 89.37 mmol/L in cold OGTT, and 280.58 ± 86.32 mmol/L in hot OGTT. Therefore, we need at least 17 subjects with 80% power and an α of 0.05. The sample size was calculated using PASS software.

## Results

### Subject characteristics

A total of 19 normal subjects and 22 newly diagnosed untreated T2DM patients completed the study. Twenty subjects (9 normal subjects and 11 patients with T2DM) had hot OGTT first and 21 had cold OGTT first. There was no significant difference in gender or age between the two groups (*P* all >0.05). The T2DM patient had a higher BMI and HbA_1c_ compared to normal subjects (Table 1).

**Table 1:** Characteristics of subjects in different groups.

	Normal	T2DM	*P* value
*N*	19	22	–
Gender (male%)	47.37%	68.18%	0.216
Age (year)	38.95 ± 2.55	44.41 ± 1.73	0.077
BMI (kg/m^2^)	22.33 ± 0.59	26.02 ± 0.61	<0.001
HbA1c (%)	5.28 ± 0.08	6.93 ± 0.28	<0.001

*T2DM* type 2 diabetes, *N* number, *BMI* body mass index, *HbA1c* glycated hemoglobin.

### Changes after hot and cold OGTT

**Fig. 1:**
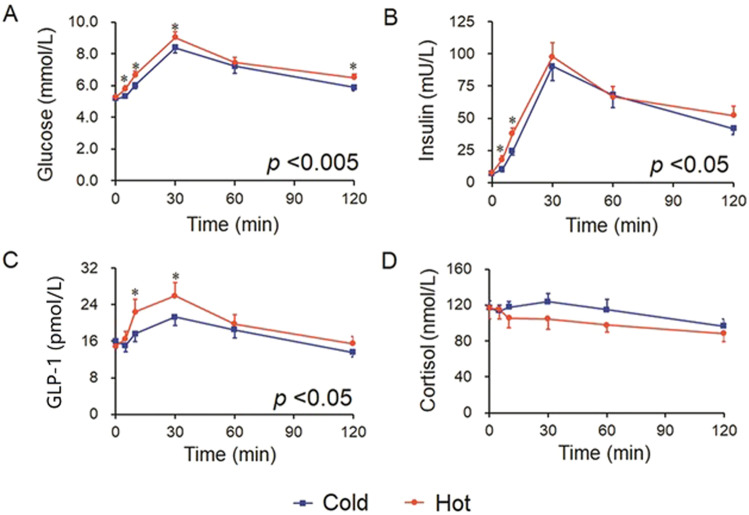
The blood glucose and glucose-responsive hormone levels in cold and hot OGTTs in normal subjects. **A** Blood glucose, **B** insulin, **C** GLP-1, and **D** cortisol levels in OGTTs in normal subjects (*n* = 19). Data were analyzed with ANOVA for repeated measurements, and all “*P*” values are from the ANOVA. If there was statistical significance between the two temperatures (*P* < 0.05), this was followed with a Bonferroni test for specific time points. Blue line=cold OGTT; red line=hot OGTT; *P < 0.05 in Bonferroni test. OGTT oral glucose-tolerance test, GLP-1 glucagon-like peptide-1.

**Fig. 2:**
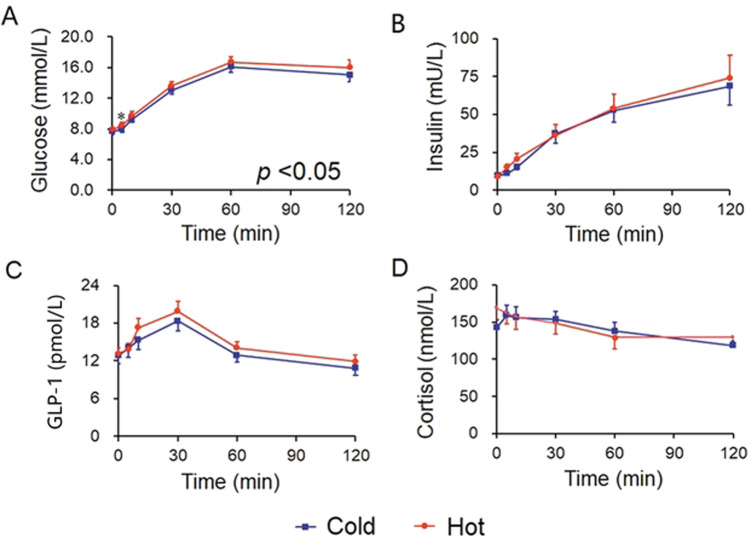
The blood glucose and glucose-responsive hormone levels in cold and hot OGTTs in patients with T2DM. **A** Blood glucose, **B** insulin, **C** GLP-1, and **D** cortisol levels in OGTTs in patients with newly diagnosed treatment naïve T2DM (*n* = 22). Data were analyzed with ANOVA for repeated measurements, and all “*P*” values are from the ANOVA. If there was statistical significance between the two temperatures (*P* < 0.05), this was followed with a Bonferroni test for specific time points. Blue line=cold OGTT; red line=hot OGTT; *P < 0.05 in Bonferroni test. OGTT oral glucose-tolerance test, GLP-1 glucagon-like peptide-1.

Table 2 shows the mean (±SE) values for each variable after hot versus cold OGTT after comparison with ANOVA for repeated measures. The mean blood glucose was significantly higher after a hot OGTT compared to a cold OGTT in both groups (*P* = 0.002 and 0.028, respectively, Table 2, Figs. 1A and 2A). Insulin and GLP-1 were also significantly higher after hot OGTT compared to cold OGTT, but only in normal subjects (both *P* < 0.05), and not in the T2DM patients (both *P* > 0.05, Table 2, Figs. 1B, C and 2B, C). The differences between the hot and cold post-OGTT responses in cortisol (Figs. 1D and 2D) and GIP (data not shown) did not reach statistical significance in both groups.

**Table 2:** Measurements after cold and hot OGTTs with ANOVA (repeated measures).

		Cold OGTT	Hot OGTT	*P* value	Increment in cold OGTT (%)	Increment in hot OGTT (%)	*P* value
Glucose (mmol/L)	Normal	6.33 ± 0.15	6.79 ± 0.14	0.002	122.46 ± 2.21	129.17 ± 2.53	0.015
	T2DM	11.35 ± 0.51	11.88 ± 0.56	0.007	151.42 ± 2.24	155.27 ± 3.51	0.104
Insulin (mU/L)	Normal	40.34 ± 3.00	46.73 ± 3.90	0.028	784.07 ± 228.61	621.54 ± 65.79	0.479
	T2DM	32.33 ± 4.71	34.81 ± 6.03	0.317	359.37 ± 34.89	400.41 ± 45.13	0.175
GLP-1 (pmol/L)	Normal	16.99 ± 1.34	19.19 ± 1.84	0.047	111.36 ± 7.56	130.20 ± 5.86	0.009
	T2DM	14.46 ± 1.04	15.40 ± 0.89	0.219	115.11 ± 5.59	117.41 ± 6.02	0.783
GIP (pmol/L)	Normal	72.05 ± 6.77	68.48 ± 5.43	0.138	111.80 ± 5.33	116.71 ± 8.71	0.621
	T2DM	52.98 ± 5.03	50.74 ± 5.04	0.327	113.38 ± 4.55	111.26 ± 6.23	0.731
Cortisol (nmol/L)	Normal	113.96 ± 4.78	104.8 ± 8.67	0.220	103.89 ± 6.04	94.47 ± 4.40	0.159
	T2DM	144.47 ± 9.19	149.17 ± 12.70	0.619	105.76 ± 544	92.95 ± 4.55	0.087

*OGTT* oral glucose-tolerance test, *GLP-1* glucagon-like peptide-1, *GIP* gastric inhibitory polypeptide, *T2DM* type 2 diabetes mellitus, *BMI* body mass index.

Data are mean ± SEM.

Further analysis of the differences in individual time points was made if the ANOVA for repeated measurements in each group was statistically significant. The blood glucose levels were higher in hot OGTT than in cold OGTT at 5, 10, 30, and 120 min in the normal group, as well as at 5 min in the T2DM group (*P* all <0.05, Figs. 1A and 2A). Insulin levels were also higher in hot OGTT than in cold OGTT at the early time points of 5 and 10 min in the normal group (*P* = 0.002 and *P* = 0.027, respectively, Fig. 1B). Statistically significant differences in GLP-1 between hot and cold OGTTs were found at 10 and 30 min in normal subjects (*P* = 0.037 and 0.023, Fig. 1C). These differences in insulin and GLP-1 between OGTTs were absent in patients with T2DM (*P* all >0.05, Fig. 2B, C).

**Fig. 3:**
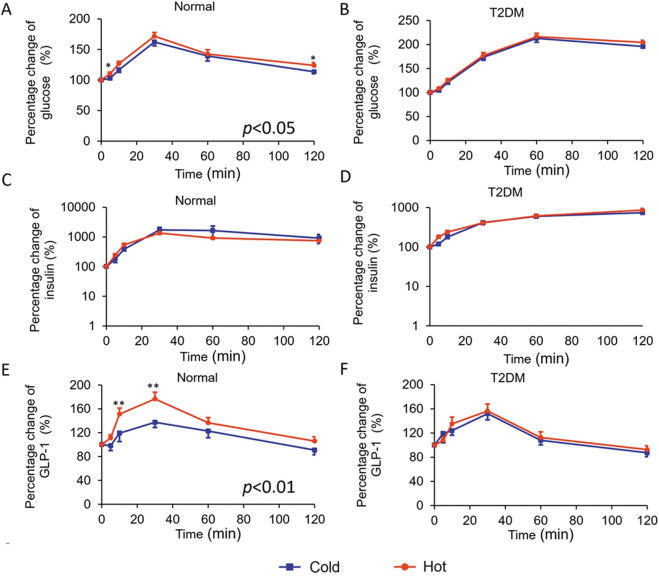
Percentage change of blood glucose and glucose-responsive hormones in cold and hot OGTTs. Percentage changes from the baseline of OGTT were also calculated as follows: values at each time point/values at baseline × 100%). The percentage change of blood glucose (**A**, **B**), insulin (**C**, **D**), and GLP-1 (**E**, **F**) with OGTTs in normal (*n* = 19) and T2DM (*n* = 22) groups. Data were analyzed with ANOVA for repeated measurements. If there was statistical significance between the two temperatures (*P* < 0.05 ANOVA), this was followed with a Bonferroni test for specific time points. Blue line=cold OGTT; red line=hot OGTT; *P < 0.05 in Bonferroni test. OGTT oral glucose-tolerance test, GLP-1 glucagon-like peptide-1, T2DM type 2 diabetes mellitus.

We also expressed the changes in blood glucose, insulin, GLP-1, GIP, and cortisol levels as a percentage of the individual baseline level for each individual participant. The incremental blood glucose levels remained higher in hot OGTT than in cold OGTT, as well as the incremental GLP-1 levels in normal subjects (*P* = 0.015 and 0.009, respectively, Table 2). Percentage change of blood glucose showed a significant elevation in hot OGTT compared with cold OGTT at 5 and 120 min in the normal group, as well as GLP-1 at 10 to 30 min (*P* all <0.05, Fig. 3A, E). However, these differences were absent in patients with T2DM (both *P* > 0.05, Fig. 3B, F). The percentage changes in insulin were similar in cold and hot OGTTs in both groups (*P* all >0.05, Fig. 3C, D).

### Factors affecting the difference in GLP-1 response between cold and hot OGTTs

We further investigated factors that may influence the glucose and GLP-1 response between cold and hot OGTTs (ΔiAUC glucose/GLP-1 = iAUC for glucose/GLP-1 in hot OGTT minus that in cold OGTT). We performed a stepwise linear regression analysis for each group separately, and for all 41 participants, with age, sex, BMI, HbA_1c_, and difference of temperature between cold and hot OGTTs as independent variables. However, none of these other factors had a statistically significant correlation with ΔiAUC glucose or GLP-1.

### Glycemic variation during hot and cold food intake days

The data of CGM also showed a statistically significant higher MBG in hot OGTT plus hot food and water, compared with cold OGTT with room temperature food and water throughout the day in both normal and diabetes groups (*P* = 0.021 and 0.022, respectively, Table 3). The SDBG in the hot food intake day was significantly higher than in cold food intake day only in the normal group (*P* = 0.043) and was absent in the diabetes group (*P* = 0.393, Table 3). However, there was no statistically significant difference of CV, TIR, and LAGE between the hot and cold food intake days in both groups (*P* all >0.05, Table 3).

**Table 3:** Glycemic variability in CGM during hot and cold food intake days.

	Normal			Type 2 diabetes		
	Cold	Hot	*P* value	Cold	Hot	*P* value
MBG (mmol/L)	5.84 ± 0.11	6.11 ± 0.13	0.021	8.46 ± 0.38	8.88 ± 0.39	0.022
SDBG (mmol/L)	0.48 ± 0.05	0.59 ± 0.06	0.043	1.87 ± 0.22	1.98 ± 0.20	0.393
CV (%)	8.11 ± 0.77	9.45 ± 0.92	0.106	21.59 ± 2.12	21.66 ± 1.87	0.962
TIR (%)	100 (100,100)	100 (100,100)	0.180	78.23 ± 4.87	74.57 ± 5.39	0.211
LAGE (mmol/L)	2.43 ± 0.29	2.75 ± 0.27	0.063	8.40 ± 0.87	8.59 ± 0.79	0.849

*MBG* the 24‐h mean glucose, *SDBG* standard deviation of the MBG, *CV* coefficient of variation, *LAGE* 24‐h largest amplitude of glycemic excursions, *TIR* time in range.

Data are mean ± SEM or median (IQR).

## Discussion

We have demonstrated for the first time that hot food may give small but statistically significant higher post-OGTT blood glucose levels compared to cold food in both normal subjects and in untreated newly diagnosed T2DM patients. We have also shown for the first time that insulin and GLP-1 levels increased significantly more with a hot OGTT in normal subjects, and this effect is lost or deficient in newly diagnosed patients with T2DM.

The differences in glucose levels were small, but statistically significant and consistently demonstrated in the OGTT and the 24-h CGM. These small differences are unlikely to make significant differences in the diagnosis of diabetes using the OGTT, but do suggest the use of a fasting glucose level as a more consistent and reliable method of diagnosis of diabetes as it would be unaffected by temperature differences. Interestingly, Booth et al. showed that each 10 °C increase in mean 30-day temperature was associated with 1.06 times higher odds of gestational diabetes mellitus [14].

The difference in GLP-1 after OGTT is interesting and novel. If the temperature of ambient glucose and presumably, other foods, does affect the GLP-1 response, it may account for some of the discrepancies in the reports on GLP-1 in normal versus diabetes. When the GLP-1 agonists and DPP-4 inhibitors were first introduced, there were differences in the reports on whether patients with T2DM had lower GLP-1 levels compared to normal non-diabetic subjects [15–17]. As far as we can discern, all these studies did not have stringent controls on the ambient temperature of the ingested glucose or meals. Our present novel finding suggests that many of these studies may need to be controlled for the ambient temperature of ingestion.

Both glucotoxicity and hyperinsulinemia are known risk factors for type 2 diabetes and obesity [18–20]. Our study has some interesting possible implications, even though these remain speculative at this stage. In countries where fast food is commonly eaten, cold sugary beverages may raise the blood glucose less that hot sugary beverages, like hot tea or coffee! (Although of course, the advice should be to avoid the use of refined sugar in all drinks! [21–24]). In China and many other countries at a similar stage of economic development, with the increase in GDP, higher consumption of sugar-sweetened beverages has become more common [25, 26]. Intake of sugar-sweetened beverages was estimated to be responsible for 0.5 million diabetes new cases in 2011 [26]. Soft drinks are usually consumed at cold temperatures in the summer, while sweetened-milk tea/coffee is usually consumed hot throughout the year. Our study may suggest the possibility that it may be preferable to choose sweet drinks that are cold, although this would need much further study!

The physiological mechanisms of hot OGTT leading to higher blood glucose are unclear. One reason may be that gastric emptying with cold drinks may be significantly slower than with hot drinks [10, 11]. Sun et al. found that both cold (4 °C) and hot (50 °C) drinks appeared to empty more slowly from the stomach than the control drink (37 °C) [10, 11]. However, cold drinks took longer than hot drinks to return to body temperature after ingestion, and this process took 30 min. Moreover, the differences in the amounts of cold and control drinks remaining in the stomach were significant up to 10 min after ingestion, which were not observed between hot and control drinks [10]. A previous study also demonstrated that the blood glucose at 30 min is related directly to the rate of gastric emptying in healthy subjects [27, 28]. In this study, the differences in blood glucose and GLP-1 levels of cold and hot OGTTs were also significant up to 30 min after ingestion, which was in keeping with the gastric emptying. Charles et al. found that after an OGTT, glucose, and insulin were elevated in a hot environment (43 °C) compared with both the cold (7.2 °C) and the neutral (22 °C) environments [29]. They postulated that reduced plasma glucose in the cold was the result of an increase in glucose uptake by shivering muscles [29, 30]. Cold exposure stimulates the sympathetic nervous system and muscle glucose uptake [31]. Moreover, the temperature of drinks or the environment may cause changes in the regional perfusion of the forearm, with subsequent changes in hormone degradation and local concentration in the venous blood. Our findings were opposite to the study in rats from Shinozaki et al. [6], who found that blood glucose was lower within 15 min after warm glucose solution administration in rats. Their findings may be confounded by the presence of anesthesia, which may affect the nervous system and muscle shivering.

Others have shown that the ambient temperature of drinks and food may influence our perception of sweetness [32–34]. Green et al. showed that cooling to 5–12 °C can reduce the perceived sweetness intensity of a glucose solution, and decrease the response of TRPM5 in the sweet taste receptor (STR) transduction cascade [34]. It is debated whether the STR triggers GLP-1 and GIP secretion. The STR was expressed in L cells in the gastrointestinal tract [35, 36], and STR blocker lactisole attenuated GLP-1 secretion response to intragastric and intraduodenal glucose infusion in humans [37]. However, the activation of STR in isolated perfused rat small intestine does not drive GIP/GLP-1 secretion [38]. Previous studies also found that GLP-1 was locally synthesized in the taste bud cells in the tongue and GLP-1 receptor existed on the gustatory nerves. The paracrine GLP-1 signaling is involved in the perception of sweet [39, 40]. Therefore, the reduction of GLP-1 levels with a cold OGTT may be a direct or indirect effect of the lower stimulation on STR in the gut or taste bud cells, which deserves further study.

Our study has several potential limitations. The study should have included a third OGTT with a body temperature or room temperature as control. However, we canceled the third OGTT for security reasons that three consecutive OGTTs without hypoglycemic treatment may lead to severe hyperglycemia in patients with diabetes. The most optimal temperature of food for blood glucose control and GLP-1 secretion should be further studied. Moreover, we could not exclude the effects of temperature on the oral cavity and salivary glands, the glucose solution should probably be given intragastrically to avoid orosensory effects in a further study.

In summary, compared with cold glucose solution, hot glucose solution increased the blood glucose, blood insulin levels, and GLP-1 levels. In patients with type 2 diabetes, GLP-1 response in hot OGTT was diminished for as yet unknown reasons.

## Data Availability

The data that support the findings of this study are available from the corresponding author upon reasonable request.

## References

[1] Jakubowicz D, Wainstein J, Ahren B, Landau Z, Bar-Dayan Y, Froy O (2015). Fasting until noon triggers increased postprandial hyperglycemia and impaired insulin response after lunch and dinner in individuals with type 2 diabetes: a randomized clinical trial. Diabetes Care.

[2] Robinson E, Almiron-Roig E, Rutters F, de Graaf C, Forde CG, Tudur Smith C (2014). A systematic review and meta-analysis examining the effect of eating rate on energy intake and hunger. Am J Clin Nutr.

[3] Hsieh SD, Muto T, Murase T, Tsuji H, Arase Y (2011). Eating until feeling full and rapid eating both increase metabolic risk factors in Japanese men and women. Public Health Nutr.

[4] Sonia S, Witjaksono F, Ridwan R (2015). Effect of cooling of cooked white rice on resistant starch content and glycemic response. Asia Pac J Clin Nutr.

[5] Nadine Najjar NA, Nahla H (2004). Glycemic and insulinemic responses to hot vs cooled potato in males with varied insulin sensitivity. Nutr Res.

[6] Shinozaki K, Shimizu Y, Shiina T, Morita H, Takewaki T (2008). Relationship between taste-induced physiological reflexes and temperature of sweet taste. Physiol Behav.

[7] Caterina MJ, Schumacher MA, Tominaga M, Rosen TA, Levine JD, Julius D (1997). The capsaicin receptor: a heat-activated ion channel in the pain pathway. Nature.

[8] Horowitz M, Edelbroek MA, Wishart JM, Straathof JW (1993). Relationship between oral glucose tolerance and gastric emptying in normal healthy subjects. Diabetologia.

[9] Schirra J, Katschinski M, Weidmann C, Schafer T, Wank U, Arnold R (1996). Gastric emptying and release of incretin hormones after glucose ingestion in humans. J Clin Invest.

[10] Sun WM, Houghton LA, Read NW, Grundy DG, Johnson AG (1988). Effect of meal temperature on gastric emptying of liquids in man. Gut.

[11] Sun WM, Penagini R, Hebbard G, Malbert C, Jones KL, Emery S (1995). Effect of drink temperature on antropyloroduodenal motility and gastric electrical activity in humans. Gut.

[12] Defronzo RA, Tripathy D, Schwenke DC, Banerji M, Bray GA, Buchanan TA (2013). Prediction of diabetes based on baseline metabolic characteristics in individuals at high risk. Diabetes Care.

[13] Lu J, Ma X, Zhou J, Zhang L, Mo Y, Ying L (2018). Association of time in range, as assessed by continuous glucose monitoring, with diabetic retinopathy in type 2 diabetes. Diabetes Care.

[14] Booth GL, Luo J, Park AL, Feig DS, Moineddin R, Ray JG (2017). Influence of environmental temperature on risk of gestational diabetes. CMAJ.

[15] Holst JJ, Knop FK, Vilsboll T, Krarup T, Madsbad S (2011). Loss of incretin effect is a specific, important, and early characteristic of type 2 diabetes. Diabetes Care.

[16] Meier JJ, Nauck MA (2010). Is the diminished incretin effect in type 2 diabetes just an epi-phenomenon of impaired beta-cell function?. Diabetes.

[17] Toft-Nielsen MB, Damholt MB, Madsbad S, Hilsted LM, Hughes TE, Michelsen BK (2001). Determinants of the impaired secretion of glucagon-like peptide-1 in type 2 diabetic patients. J Clin Endocrinol Metab.

[18] Zimmet PZ, Collins VR, Dowse GK, Knight LT (1992). Hyperinsulinaemia in youth is a predictor of type 2 (non-insulin-dependent) diabetes mellitus. Diabetologia.

[19] Ottosson-Laakso E, Krus U, Storm P, Prasad RB, Oskolkov N, Ahlqvist E (2017). Glucose-induced changes in gene expression in human pancreatic islets: causes or consequences of chronic hyperglycemia. Diabetes.

[20] Stumvoll M, Goldstein BJ, van Haeften TW (2005). Type 2 diabetes: principles of pathogenesis and therapy. Lancet.

[21] Fardet A, Boirie Y (2014). Associations between food and beverage groups and major diet-related chronic diseases: an exhaustive review of pooled/meta-analyses and systematic reviews. Nutr Rev.

[22] Imamura F, O’Connor L, Ye Z, Mursu J, Hayashino Y, Bhupathiraju SN (2016). Consumption of sugar sweetened beverages, artificially sweetened beverages, and fruit juice and incidence of type 2 diabetes: systematic review, meta-analysis, and estimation of population attributable fraction. Br J Sports Med.

[23] O’Connor L, Imamura F, Lentjes MA, Khaw KT, Wareham NJ, Forouhi NG (2015). Prospective associations and population impact of sweet beverage intake and type 2 diabetes, and effects of substitutions with alternative beverages. Diabetologia.

[24] Basu S, McKee M, Galea G, Stuckler D (2013). Relationship of soft drink consumption to global overweight, obesity, and diabetes: a cross-national analysis of 75 countries. Am J Public Health.

[25] Zhang N, Du SM, Ma GS (2017). Current lifestyle factors that increase risk of T2DM in China. Eur J Clin Nutr.

[26] Li Y, Wang DD, Ley SH, Vasanti M, Howard AG, He Y (2017). Time trends of dietary and lifestyle factors and their potential impact on diabetes burden in China. Diabetes Care.

[27] Marathe CS, Rayner CK, Lange K, Bound M, Wishart J, Jones KL (2017). Relationships of the early insulin secretory response and oral disposition index with gastric emptying in subjects with normal glucose tolerance. Physiol Rep.

[28] Marathe CS, Horowitz M, Trahair LG, Wishart JM, Bound M, Lange K (2015). Relationships of early and late glycemic responses with gastric emptying during an oral glucose tolerance test. J Clin Endocrinol Metab.

[29] Dumke CL, Slivka DR, Cuddy JS, Hailes WS, Rose SM, Ruby BC (2015). The effect of environmental temperature on glucose and insulin after an oral glucose tolerance test in healthy young men. Wilderness Environ Med.

[30] Vallerand AL, Frim J, Kavanagh MF (1988). Plasma glucose and insulin responses to oral and intravenous glucose in cold-exposed humans. J Appl Physiol.

[31] Blondin DP, Labbe SM, Phoenix S, Guerin B, Turcotte EE, Richard D (2015). Contributions of white and brown adipose tissues and skeletal muscles to acute cold-induced metabolic responses in healthy men. J Physiol.

[32] Green BG, Frankmann SP (1988). The effect of cooling on the perception of carbohydrate and intensive sweeteners. Physiol Behav.

[33] Calvino AM (1986). Perception of sweetness: the effects of concentration and temperature. Physiol Behav.

[34] Green BG, Nachtigal D (2015). Temperature affects human sweet taste via at least two mechanisms. Chem Senses.

[35] Feng R, Qian C, Liu Q, Jin Y, Liu L, Li S (2017). Expression of sweet taste receptor and gut hormone secretion in modelled type 2 diabetes. Gen Comp Endocrinol.

[36] Kokrashvili Z, Mosinger B, Margolskee RF (2009). Taste signaling elements expressed in gut enteroendocrine cells regulate nutrient-responsive secretion of gut hormones. Am J Clin Nutr.

[37] Gerspach AC, Steinert RE, Schonenberger L, Graber-Maier A, Beglinger C (2011). The role of the gut sweet taste receptor in regulating GLP-1, PYY, and CCK release in humans. Am J Physiol Endocrinol Metab.

[38] Saltiel MY, Kuhre RE, Christiansen CB, Eliasen R, Conde-Frieboes KW, Rosenkilde MM (2017). Sweet taste receptor activation in the gut is of limited importance for glucose-stimulated GLP-1 and GIP secretion. Nutrients.

[39] Jensterle M, DeVries JH, Battelino T, Battelino S, Yildiz B, Janez A (2021). Glucagon-like peptide-1, a matter of taste?. Rev Endocr Metab Disord.

[40] Calvo SS, Egan JM (2015). The endocrinology of taste receptors. Nat Rev Endocrinol.

